# Sea spiders (Arthropoda, Pycnogonida) from ten recent research expeditions to the Antarctic Peninsula, Scotia Arc and Weddell Sea - data

**DOI:** 10.3897/BDJ.10.e79353

**Published:** 2022-06-14

**Authors:** Jamie Maxwell, Yi Ming Gan, Claudia Arango, Jana S Doemel, A. Louise Allcock, Anton P. van de Putte, Huw Griffiths

**Affiliations:** 1 National University of Ireland, Galway, Galway, Ireland National University of Ireland, Galway Galway Ireland; 2 Royal Belgian Institute of Natural Sciences, Brussels, Belgium Royal Belgian Institute of Natural Sciences Brussels Belgium; 3 Queensland Museum, Brisbane, Australia Queensland Museum Brisbane Australia; 4 University of Duisburg-Essen, Essen, Germany University of Duisburg-Essen Essen Germany; 5 British Antarctic Survey, Cambridge, United Kingdom British Antarctic Survey Cambridge United Kingdom

**Keywords:** occurrence, abundance, Southern Ocean, biodiversity, epifauna

## Abstract

**Background:**

This dataset contains information on specimens of Southern Ocean Pycnogonida (Arthropoda), that were collected from ten different research cruises, spanning 13 years. The individual aims and objectives of each cruise can be found in their cruise reports. The specimens have been collated into a single dataset, forming the basis of J. Maxwell’s PhD. The dataset will be used to investigate the community structure of Antarctic pycnogonids and the factors which influence its composition. This dataset is published by SCAR-AntOBIS under the licence CC-BY 4.0. Please follow the guidelines from the SCAR and IPY Data Policies (https://www.scar.org/excom-meetings/xxxi-scar-delegates-2010-buenos-aires-argentina/4563-scar-xxxi-ip04b-scar-data-policy/file/) when using the data. If you have any questions regarding this dataset, please do not hesitate to contact us via the contact information provided in the metadata or via data-biodiversity-aq@naturalsciences.be.

**New information:**

This dataset adds vital occurrence and abundance data for pycnogonids from 10 previously unexamined research cruises from the Weddell Sea, Antarctic Penisula and the islands of the Scotia Arc. It includes the first pycnogonid data from the Prince Gustav Channel. The 197 sampling stations within this dataset represent an 11% increase in the number of stations where pycnogonids have been recorded in the Southern Ocean, southern South America and New Zealand waters and an 18% increase for above 60 degrees latitude. Presence data for any observed epifauna are also included.

## Introduction

Pycnogonida, or sea spiders, are a class of Arthropoda found throughout the marine realm and are a sister group to the Euchelicerata (Ballesteros et al. 2021). Globally, there are over 1,300 described species from ten different families. Although pycnogonids are a cosmopolitan marine taxon, the Southern Ocean is particularly rich in terms of species, with around 20% of all known species found here and 14% found south of the Polar Front in Antarctic waters (Griffiths et al. 2011). With this high diversity, there is also a high degree of endemism, with 40% of the reported Antarctic species considered to be endemic (Soler-Membrives et al. 2014). Uniquely, Southern Ocean waters are home to representatives of all ten pycnogonid families (Munilla and Soler Membrives 2009), which has led to the hypothesis that the Antarctic is their evolutionary centre of origin (Stock 1957, Hedgpeth 1969, León 2001, Griffiths et al. 2011).

Antarctic pycnogonids have been studied for nearly two centuries. Most of this research has concentrated on taxonomic work and species descriptions, research which continues today with new species being described regularly (Cano Sánchez and López-González 2013, Cano-Sánchez and López-González 2018, Cano-Sánchez and López-González 2019, Dömel et al. 2019). The most comprehensive and up-to-date species list is by Munilla and Soler Membrives (2009). Pycnogonids are well represented within the online portal the Global Biodiversity Information Facility (OBIS 2022). When these databases are filtered for Pycnogonida, presence only, below -40 degrees, GBIF returns 14,086 records, while OBIS has 11,655. These records include duplicate records and specimens not identified to species level. The largest single database of pycnogonids within both GBIF and OBIS is SOMBASE Pycnogonids (British Antarctic Survey 2022), which is based on the records from over 100 years of literature. It contains 7399 occurrence records and 1837 sample locations of pycnogonids from the Southern Ocean and the neighbouring regions ((GBIF 2022) and the Ocean Biodiversity Information System (OBIS 2022)).

Despite relatively good taxonomic knowledge, little is understood about the community structure of Southern Ocean pycnogonids. The majority of investigations into pycnogonid community structure have focused on localised distribution (Munilla and Soler-Membrives 2007, Nielsen et al. 2009, Soler i Membrives et al. 2009, Munilla and Soler-Membrives 2015), continent-wide studies undertaken at a coarse resolution (Griffiths et al. 2011, Soler-Membrives et al. 2014) or are included within larger benthic community studies (San Vicente et al. 1997, Griffiths et al. 2009) . Species richness has been shown to decrease with depth, with more taxa on the shelf than slope (Munilla and Soler-Membrives 2007, Soler i Membrives et al. 2009), but with most species on the shelf having a wide bathymetric range (Griffiths et al. 2011). This is possibly due to past retreats into deep-sea refugia during glacial periods before recolonising the shelf.

Ocean-wide studies have highlighted the Eastern Weddell Sea and Bransfield Strait as possible diversity hotspots (Soler-Membrives et al. 2014), though it is important to consider the challenges of sampling bias, particularly around the well-studied West Antarctic Peninsula which appears to be highly diverse. Over 20% of species are reported to have circumpolar distributions (León 2001). Molecular studies have shown that some species, for example, *Nymphonaustrale* Hodgson, 1902, is a single circumpolar species, but with some population structure related to geographic distance (Arango et al. 2011, Collins et al. 2018). Molecular analysis has also uncovered species complexes and cryptic species (Krabbe et al. 2010, Dietz et al. 2013, Weis et al. 2014).

This study aimed to increase the distributional knowledge of Southern Ocean pycnogonids, in particular those found south of 60 degrees around the Antarctic Peninsula, Scotia Arc and Weddell Sea, through the examination and identification of over 5,000 previously-unstudied specimens, the results of which are presented here. These new data will be used, in conjunction with previously available data, to analyse the community structure of the pycnogonids and the factors which may drive their distribution.

## Project description

### Title

Sea spiders (Arthropoda, Pycnogonida) from ten recent research expeditions to the Antarctic Peninsula, Scotia Arc and Weddell Sea - data

### Personnel

Jamie Maxwell, Claudia Arango, Jana Dömel, Huw Griffiths, Louise Allcock, Yi-Ming Gan

### Funding

Irish Research Council Postgraduate Scholarship GOIPG/2019/4020. The publication of this data paper was supported by the SCAR Antarctic Biodiversity Portal (Biodiversity.aq), one of Belgium’s contributions to EU Lifewatch.

## Sampling methods

### Study extent

The pycnogonids in this study were collected during ten research cruises, over 13 years, in the area between 110°W - 5°E and 50°N - 78°S. The samples were collected from different areas in the Southern Ocean, mainly the Weddell sea, South Orkney Islands and the Western Antarctic Peninsula. Sampling took place during ten expeditions on the RRS *James Clark Ros*s, RV *Polarstern* and RRS *Discovery*. In total, 197 stations sampled contained at least one pycnogonid. Sampling took place between 2007 and 2019. For the full list of cruises and their details, see Table 1.

### Sampling description

Sampling methods

Most specimens were sampled using an Agassiz trawl (AGT) or an epibenthic sledge (EBS) with 165 AGT and 14 EBS deployments. The AGT had a mesh size of 1 cm and a mouth width of 2 m (except for JR17007 where the mouth was 1.25 m). AGTs were deployed to a depth of between 54 and 2279 m.

The EBS had a suprabenthic and an epibenthic net, both with a mesh size of 500 μm (cod-ends 300 μm). The epibenthic net extended from 27 cm to 60 cm above the bottom, with the suprabenthic net extending from 100 cm to 133 cm. The EBS was deployed as described by Brenke (2005) and was fitted with an open-closing mechanism so that the mouths of both nets were closed whenever the EBS was not in contact with the seafloor. The EBS was deployed to depths of between 436 and 5339 m. Both the AGT and EBS were deployed for approximately 10 minutes (trawling time) and at a speed of 1 knot.

Pycnogonids were also recovered from Rauschert dredge (RD) deployments, once during PS118 and on two occasions during both JR15005 and PS77. During PS118 and PS77, the RD mesh size was 1 mm, while a mesh of 500 μm was used on JR15005. The RD was deployed, attached to the AGT with 5 metres of cable. Deployments were between 278.5 – 817 m.

A bottom trawl (BT) was used during PS77, which was a 130 ft trawl with a 10 mm herring cod-end. This was deployed 11 times at depths of between 223.5 and 486 m. The protocol for the deployment of the BT was similar to that of the AGT, but with slightly longer trawl times (approx. 20 min).

A single pycnogonid was recovered from a kelp raft that was recovered from the surface during JR15005.

### Quality control

All records were validated. - Coordinates were plotted to verify the geographical location and locality. - All scientific names were checked for typos and matched to the species information backbone of Worlds Register of Marine Species (http://marinespecies.org/) and LSID were assigned to each taxon as scientificNameID. - Event date and time were converted into ISO 8601.

### Step description

Sample Processing on deck

EBS – Once the gear was returned to the deck, samples were sieved (300 μm) and/or transferred into pre-cooled (-20°C) 96% ethanol, which was then stored at -20°C for at least 48 hours before further processing to avoid DNA degradation. After at least 48 hours, samples were sorted to the lowest taxonomic level possible, counted and stored in 96% ethanol.

AGT and BT – Once on deck, samples were sorted to the lowest taxonomic level possible, counted, placed in pre-cooled 96% ethanol and stored at -20°C. During the RRS *James Clark Ross* cruises, all specimens recovered in the trawl were preserved. For the other cruises on the RRS *Discovery* and RV *Polarstern*, preservation was done as described; however, it could not be verified that all specimens were recovered from each trawl as sampling may have been selective.

RD - The on-deck protocol for the RD was the same as for the EBS, unless specimens were large and immediately obvious, in which case these were separated straight away and transferred to pre-cooled 96% ethanol and stored at -20°C.

The kelp sample (Jr15005) was immediately sorted to the lowest taxonomic level possible, counted and stored in pre-cooled 96% ethanol.

Specimens remained in -20°C storage until returned to the UK. Once specimens were returned to British Antarctic Survey in Cambridge, they were stored at ambient temperature.

Treatment of Samples

Every specimen was examined using a stereoscope and identified to the lowest taxonomic rank possible, using taxonomic keys and original descriptions (e.g. Hodgson 1907, Gordon 1932, Gordon 1944, Fry and Hedgpeth 1969, Child 1994a, Child 1994b, Child 1995a, Child 1995b, Child 1995c). Most specimens (5498) were identified by J. Maxwell at the University of Ireland Galway, with 159 individuals identified by C. Arango and an additional 50 identified by J. Domel. The online portal World Registry of Marine Species (WoRMS) was used to confirm acceptance of species names and the online Biodiversity Heritage Library was used to source many of the original descriptions. Where identification was inconclusive, only genus or family names were assigned. The nomenclature used for these specimens followed Horton et al. (2021).

To further aid in identification, tissue samples were sent to BOLD to be barcoded using the COI-5P region of the cytochrome c oxidase subunit I gene. These will be made publicly available in the future, but if not available at the time of reading, they can be requested from the corresponding author (BOLD Project - NUIG Untangling the Sea Spider's Web: Investigating the Biogeography and Evolutionary History of Pycnogonida).

Any epifauna easily observable on individual pycnogonids was noted and identified to the lowest taxonomic rank possible. As the epifauna was not the focus of the project, the identification was rarely lower than Order and only occurrence was recorded. All epifauna were preserved together with the associated host.

All the BAS samples are on a long term loan to J. Maxwell. All samples from DY099 are stored in the Natural History Museum London. The data were uploaded to Global Biodiversity Information Facility (Maxwell et al. 2021)

## Geographic coverage

### Description

**Figure 1.**
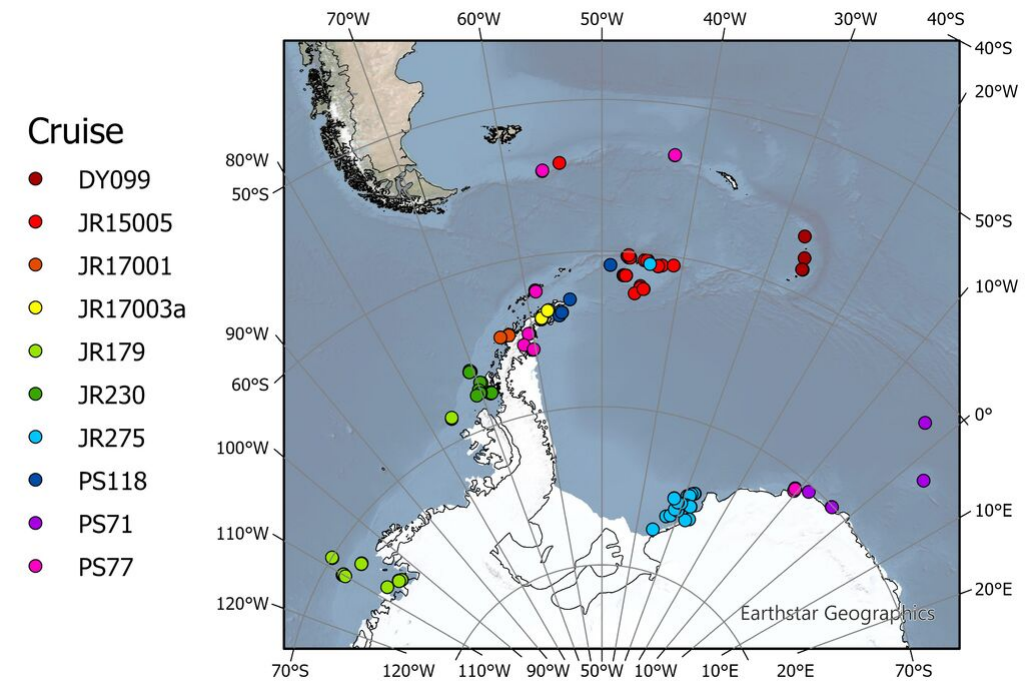
The location of the sampling stations and the cruises during which they were conducted. For cruise details and cruise reports, see Table 1.

The samples were collected from different areas in the Southern Ocean, mainly the Weddell sea, South Orkney Islands and the Western Antarctic Peninsula (Fig. 1). The 197 sampling stations within this dataset represent an 11% increase in the number of stations where pycnogonids have been recorded in the Southern Ocean, southern South America and New Zealand waters (Soler-Membrives et al. 2014).

### Coordinates

-77.358 and -53.398 Latitude; -110.013 and 2.881 Longitude.

## Taxonomic coverage

### Description

General taxonomic coverage description: The Pycnogonida specimens consist of eight families, 15 genera and 81 species plus 16 morphotypes which could not be identified with 100% certainty (i.e. sp. inc., gen. aff. or sp. aff.). For a full list of species see the "Taxa included" table below and for the number of individuals in each Family, genus and species, see Table 2. The 81 confirmed species in this dataset represent 30% of the species recorded in Antarctic and Sub-Antarctic waters (Munilla and Soler Membrives 2009).

The most frequently recovered species, i.e. the species reported at the most stations, was *Nymphonaustrale* Hodgson, 1902, which was recovered from 56 stations. *Pallenopsishodgsoni* Gordon 1938 was the second most abundant, found at 44 stations, followed by *Colossendeismegalonyx* Hoek, 1881 (42 stations), *Austropallenecornigera* (Möbius, 1902) (34 stations) and *Nymphonunguiculatum* Hodgson, 1915 (25 stations). Sixty-four per cent of the 197 stations had three or fewer species. For the number of species sampled on each cruise, see Table 1.

*Nymphonaustrale* was also the most numerous species in the dataset with 3,004 individuals identified. All of the five most numerous species were from the family Nymphonidae. After *N.australe*, *Nymphonbouvieri* Gordon, 1932 had 689 individuals, followed by *Nymphonproceroides* Bouvier, 1911 (376), *Nymphoneltaninae* Child, 1995 (166) and *Pentanymphonantarcticum* Hodgson, 1904 (129). Of the 97 identified taxa, 60% of them had five or fewer individuals.

Epifaunas from nine different phyla were recorded.

### Taxa included

**Table:** 

Rank	Scientific Name	
genus	*Achelia* sp. inc.	
species	*Acheliacommunis*	
species	*Acheliaspicata*	
species	*Ammotheabentartica*	
species	*Ammotheabicorniculata*	
species	*Ammotheabigibbosa*	
species	*Ammotheacalmani*	
species	*Ammotheacarolinensis*	
species	*Ammotheachildi*	
species	*Ammotheaclausi*	
species	*Ammotheagigantea*	
species	*Ammotheaglacialis*	
species	*Ammotheahesperidensis*	
species	*Ammothealongispina*	
species	*Ammotheameridionalis*	
species	*Ammotheaminor*	
species	*Ammotheaspinosa*	
species	*Ammotheastriata*	
species	*Ammotheastylirostris*	
family	Ammotheidae gen. aff	
species	*Anoplodactylusaustralis*	
species	*Austrodecusglaciale*	
species	*Austropallenebrachiura*	
species	*Austropallenecornigera*	
species	*Austropallenegracilipes*	
species	*Austropallenespinicornis*	
species	*Austropallenetenuicornis*	
species	*Austroraptusjuvenilis*	
species	*Cilunculuscactoides*	
genus	*Colossendeis*	
species	*Colossendeisaustralis*	
species	*Colossendeisbouvetensis*	
genus	*Colossendeis* sp. inc.	
species	*Colossendeisdrakei*	
species	*Colossendeisensifer*	
species	*Colossendeisglacialis*	
species	*Colossendeislongirstris*	
species	*Colossendeismegalonyx*	
species	*Colossendeisrobusta*	
species	*Colossendeisscotti*	
species	*Colossendeistortipalpis*	
species	*Dodecolopodamawsoni*	
species	*Nymphonaustrale*	
species	*Nymphonaustralecaecum*	
species	*Nymphonbanzare*	
species	*Nymphonbiarticulatum*	
species	*Nymphonbouvieri*	
species	*Nymphonbrevicaudatum*	
genus	*Nymphon* sp. inc.	
species	*Nymphoncharcoti*	
species	*Nymphoneltaninae*	
species	*Nymphonhiemale*	
species	*Nymphoninornatum*	
species	*Nymphonlanare*	
species	*Nymphonlongicoxa*	
species	*Nymphonmendosum*	
species	*Nymphonmultituberculatum*	
species	*Nymphonneumayri*	
species	*Nymphonpagophilum*	
species	*Nymphonproceroides*	
species	*Nymphonproximum*	
genus	*Nymphon* sp. indet.	
genus	*Nymphon* stet. A	
genus	*Nymphon* stet. B	
species	*Nymphonsubtile*	
species	*Nymphontenuimanum*	
species	*Nymphontenuipes*	
species	*Nymphonunguiculatum*	
species	*Nymphonvillosum*	
species	*Pallenopsisbuphtalmus*	
species	*Pallenopsisgracilis*	
species	*Pallenopsishodgsoni*	
species	*Pallenopsislatefrontalis*	
species	*Pallenopsisleiopus*	
species	*Pallenopsismacronyx*	
species	*Pallenopsisobstaculumsuperavit*	
species	*Pallenopsispatagonica*	
species	*Pallenopsispilosa*	
species	*Pallenopsisrotunda*	
genus	*Pallenopsis* sp. indet.	
genus	*Pallenopsis* indet.	
species	*Pallenopsisspicata*	
species	*Pallenopsisvanhoeffeni*	
genus	*Pallenopsis* cf.	
genus	*Pantopipetta* sp. stet.	
species	*Pentanymphonantarcticum*	
species	*Pentapycnonbouvieri*	
species	*Pentapycnoncharcoti*	
species	*Pycnogonumdiceros*	
species	*Pycnogonumgaini*	
species	*Pycnogonumgordonae*	

## Temporal coverage

**Single date:** .

### Notes

2007-12-22 through 2019-04-02

## Usage licence

### Usage licence

Other

### IP rights notes

This work is licensed under a Creative Commons Attribution (CC-BY) 4.0 License.

## Data resources

### Data package title

Sea spiders (Arthropoda, Pycnogonida) from ten recent research expeditions to the Antarctic Peninsula, Scotia Arc and Weddell Sea - data

### Resource link

https://www.gbif.org/dataset/1e7b6980-0842-4c4a-8b14-541b95d2ed3c

### Alternative identifiers

https://ipt.biodiversity.aq/resource?r=bas-pycnogonida_2007-2019, https://doi.org/10.15468/re3ffz, 1e7b6980-0842-4c4a-8b14-541b95d2ed3c

### Number of data sets

1

### Data set 1.

#### Data set name

Sea spiders (Arthropoda, Pycnogonida) from ten recent research expeditions to the Antarctic Peninsula, Scotia Arc and Weddell Sea - data

#### Data format

Darwin Core

#### Description

The dataset contains information on specimens of Southern Ocean Pycnogonida (Arthropoda) that were collected from ten different research cruises, spanning 13 years. The individual aims and objectives of each cruise can be found in their cruise reports. The specimens have been collated into a single dataset, forming the basis of J. Maxwell’s PhD. The dataset will be used to investigate the community structure of Antarctic pycnogonids and the factors which influence its composition. This dataset is published by SCAR-AntOBIS under the licence CC-BY 4.0. Please follow the guidelines from the SCAR and IPY Data Policies (https://www.scar.org/excom-meetings/xxxi-scar-delegates-2010-buenos-aires-argentina/4563-scar-xxxi-ip04b-scar-data-policy/file/) when using the data. If you have any questions regarding this dataset, please do not hesitate to contact us via the contact information provided in the metadata or via data-biodiversity-aq@naturalsciences.be.

**Data set 1.** 

Column label	Column description
id	id number.
type	specimen type, physical, molecular etc.
Language	language.
institutionID	an identifier for the institution having custody of the specimens.
institutionCode	institution code.
basisOfRecord	the specific nature of the data record.
occurrenceID	an identifier for the Occurrence/specimen.
occurrenceRemarks	notes on occurence/specimen, if any.
organismQuantity	number of individuals.
organismQuantityType	the type of quantification system used for the quantity of organisms.
sex	the sex of the biological individual(s) represented in the Occurrence.
lifeStage	the age class or life stage of the Organism(s) at the time the Occurrence was recorded.
occurrenceStatus	a statement about the presence or absence of a Taxon at a Location.
preparations	a list (concatenated and separated) of preparations and preservation methods for a specimen.
associatedMedia	a list (concatenated and separated) of identifiers (publication, global unique identifier, URI) of media associated with the Occurrence.
associatedOccurrences	a list (concatenated and separated) of identifiers of other Occurrence records and their associations with this Occurrence.
eventID	an identifier for the set of information associated with an Event (something that occurs at a place and time). This may be a global unique identifier or an identifier specific to the dataset.
samplingProtocol	gear used to collect specimens.
eventDate	the date-time or interval during which an Event occurred.
year	year.
month	month.
day	day.
verbatimEventDate	the verbatim original representation of the date and time information for an Event.
fieldNumber	field number.
eventRemarks	remarks on event, if any.
countryCode	the standard code for the country in which the Location occurs.
minimumDepthInMetres	minimum depth during event in metres.
maximumDepthInMetres	maximum depth during event in metres.
startLatitude	the start latitude of a transect.
startLongitude	the start longitude of a transect.
decimalLatitude	the geographic latitude (in decimal degrees, using the spatial reference system given in geodeticDatum) of the geographic centre of a Location. Positive values are north of the Equator, negative values are south of it. Legal values lie between -90 and 90, inclusive.
decimalLongitude	the geographic longitude (in decimal degrees, using the spatial reference system given in geodeticDatum) of the geographic centre of a Location. Positive values are east of the Greenwich Meridian, negative values are west of it. Legal values lie between -180 and 180, inclusive.
geodeticDatum	the ellipsoid, geodetic datum, or spatial reference system (SRS) upon which the geographic coordinates given in decimalLatitude and decimalLongitude are based.
coordinateUncertaintyInMetres	the horizontal distance (in metres) from the given decimalLatitude and decimalLongitude describing the smallest circle containing the whole of the Location. Empty if the uncertainty is unknown, cannot be estimated or is not applicable (because there are no coordinates).
catalogNumber	an identifier of any form assigned by the source within a physical collection or digital dataset for the record which may not be unique, but should be fairly unique in combination with the institution and collection code.
footprintWKT	a Well-Known Text (WKT) representation of the shape (footprint, geometry) that defines the Location.
identifiedBy	a list (concatenated and separated) of names of people, groups or organisations who assigned the Taxon to the subject.
identifiedByID	identifiers ORCID iD.
identificationRemarks	remarks on identification, if any.
scientificNameID	marinespecies.org taxon number.
scientificName	scientific name.
kingdom	the full scientific name of the kingdom in which the taxon is classified.
phylum	the full scientific name of the phylum in whch the taxon is classified.
class	the full scientific name of the class in which the taxon is classified.
order	the full scientific name of the order in which the taxon is classified.
family	the full scientific name of the family in which the taxon is classified.
genus	the full scientific name of the genus in which the taxon is classified.
specificEpithet	the name of the first or species epithet of the scientificName.
infraspecificEpithet	the infrageneric part of a binomial name at ranks above species, but below genus.
identificationQualifier	a brief phrase or a standard term ("cf.", "aff.") to express the determiner's doubts about the Identification.
taxonRank	the taxonomic rank of the most specific name in the scientificName.
scientificNameAuthorship	the authorship information for the scientificName formatted according to the conventions of the applicable nomenclaturalCode.

## Figures and Tables

**Table 1.** Cruise data including temporal range, gear used, number of stations and number of specimens collected. AGT = Agassiz trawl. BT = Bottom Trawl, EBS = Epibenthic sledge, RD = Rauschert dredge.

Cruise ID	Ship	Dates	# Stations	# Families	# Genera	# Species	# Individuals	Gear (# Deployments)	Cruise report	
PS71	RV Polarstern	2007/11/28 – 2008/02/04	5	4	5	11	34	AGT (5)	https://www.bodc.ac.uk/resources/inventories/cruise_inventory/reports/polarstern_antxxiv3.pdf	
JR179	RRS James Clark Ross	2008/02/21 – 2008/04/11	9	2	3	9	42	EBS (9)	https://www.bodc.ac.uk/resources/inventories/cruise_inventory/reports/jr179.pdf	
JR230	RRS James Clark Ross	2009/12/02 – 2009/12/11	29	5	6	18	159	AGT (29)	https://www.bodc.ac.uk/resources/inventories/cruise_inventory/reports/jr230.pdf	
PS77	RV Polarstern	2011/02/08 – 2011/04/18	25	6	10	38	346	AGT (11), BT (12), RD (2)	https://epic.awi.de/id/eprint/30175/1/644-2012%20ANT27-3%20RKnust.pdf	
JR275	RRS James Clark Ross	2012/02/07 – 2012/03/22	46	6	8	34	535	AGT (46)	https://www.bodc.ac.uk/resources/inventories/cruise_inventory/reports/jr275.pdf	
JR15005	RRS James Clark Ross	2016/02/26 – 2016/03/24	54	7	10	40	2686	AGT (51), RD (2), Kelp (1)	https://www.bodc.ac.uk/resources/inventories/cruise_inventory/reports/jr15005.pdf	
JR17001	RRS James Clark Ross	2017/11/21 – 2017/12/21	8	2	3	4	41	AGT (8)	https://www.bodc.ac.uk/resources/inventories/cruise_inventory/reports/jr17001.pdf	
JR17003a	RRS James Clark Ross	2018/02/23 – 2018/03/11	10	7	10	27	1518	AGT (7), EBS (3)	https://www.bodc.ac.uk/resources/inventories/cruise_inventory/reports/jr17003a.pdf	
PS118	RV Polarstern	2019/02/09 – 2019/04/10	7	6	6	13	36	AGT (6), RD (1)	https://epic.awi.de/id/eprint/48988/1/Expeditionsprogramm_PS118_Dorschel.pdf	
DY099	RRS Discovery	2019/02/15 – 2019/03/09	4	2	3	7	310	AGT (4)	https://www.bodc.ac.uk/resources/inventories/cruise_inventory/reports/dy099_research.pdf	

**Table 2.** The breakdown of the 5707 individuals in the dataset into totals for each Family, Genus, and Species.

**Family**	# **ind.**	**Genus**	# **ind.**	**Species**	# **ind.**
**Ammotheidae**	87				
		**Achelia**	5		
				*Acheliaassimilis* sp. inc.	1
				*Acheliacommunis*	3
				*Acheliaspicata*	1
		**Ammothea**	72		
				*Ammotheabentartica*	1
				*Ammotheabicorniculata*	5
				*Ammotheabigibbosa*	1
				*Ammotheacalmani*	4
				*Ammotheacarolinensis*	21
				*Ammotheachildi*	1
				*Ammotheaclausi*	7
				*Ammotheagigantea*	2
				*Ammotheaglacialis*	3
				*Ammotheahesperidensis*	6
				*Ammothealongispina*	6
				*Ammotheameridionalis*	5
				*Ammotheaminor*	2
				*Ammotheaspinosa*	2
				*Ammotheastriata*	2
				*Ammotheastylirostris*	4
		**Ammotheidae** **gen. aff**	3		
				*Ammotheidae* gen. aff	3
		**Austroraptus**	3		
				*Austroraptusjuvenilis*	3
		**Cilunculus**	4		
				*Cilunculuscactoides*	4
**Austrodecidae**	6				
		**Austrodecus**	5		
				*Austrodecusglaciale*	5
		**Pantopipetta**	1		
				*Pantopipetta* sp. stet.	1
**Callipallenidae**	134				
		**Austropallene**	134		
				*Austropallenebrachiura*	5
				*Austropallenecornigera*	93
				*Austropallenegracilipes*	2
				*Austropallenespinicornis*	27
				*Austropallenetenuicornis*	7
**Colossendeidae**	156				
		**Colossendeis**	154		
				*Colossendeis* sp. indet	1
				*Colossendeisaugusta* sp. aff.	1
				*Colossendeisaustralis*	2
				*Colossendeisavidus* sp. inc.	1
				*Colossendeisbouvetensis*	6
				*Colossendeiscolossea* sp. inc.	1
				*Colossendeisdrakei*	3
				*Colossendeisensifer*	1
				*Colossendeisglacialis*	4
				*Colossendeislilliei* sp.inc.	1
				*Colossendeislongirstris*	1
				*Colossendeismegalonyx*	114
				*Colossendeisrobusta*	2
				*Colossendeisrobusta* sp. inc.	1
				*Colossendeisscotti*	1
				*Colossendeistortipalpis*	14
		**Dodecolopoda**	2		
				*Dodecolopodamawsoni*	2
**Nymphonidae**	5019				
		**Nymphon**	4888		
				*Nymphonaustrale*	3004
				*Nymphonaustralecaecum*	13
				*Nymphonbanzare*	2
				*Nymphonbiarticulatum*	33
				*Nymphonbouvieri*	689
				*Nymphonbouvieri* sp. inc.	27
				*Nymphonbrevicaudatum*	107
				*Nymphoncharcoti*	33
				*Nymphoncharcoti* sp. inc	1
				*Nymphoncompactum* sp. inc.	1
				*Nymphondistensum* sp. inc	1
				*Nymphoneltaninae*	166
				*Nymphoneltaninae* sp. inc.	4
				*Nymphonhiemale*	30
				*Nymphoninornatum*	2
				*Nymphonlanare*	61
				*Nymphonlongicoxa*	84
				*Nymphonmendosum*	2
				*Nymphonmultiarticulatum*	5
				*Nymphonmultituberculatum*	7
				*Nymphonneumayri*	13
				*Nymphonorcadense* sp. inc	1
				*Nymphonpagophilum*	9
				*Nymphonproceroides*	376
				*Nymphonproximum*	17
				*Nymphon* sp. indet.	13
				*Nymphon* sp. stet. A	3
				*Nymphon* sp. stet. B	1
				*Nymphonsubtile*	2
				*Nymphontenuimanum*	2
				*Nymphontenuipes*	57
				*Nymphontenuipes* sp. inc.	1
				*Nymphonunguiculatum*	107
				*Nymphonunguiculatum* sp. inc.	1
				*Nymphonvillosum*	13
		**Pentanymphon**	131		
				*Pentanymphonantarcticum*	131
**Pallenopsidae**	279				
		**Pallenopsis**	279		
				*Pallenopsisbuphtalmus*	1
				*Pallenopsisgracilis*	9
				*Pallenopsishodgsoni*	104
				*Pallenopsishodgsoni* cf.	6
				*Pallenopsislatefrontalis*	13
				*Pallenopsisleiopus*	1
				*Pallenopsismacronyx*	57
				*Pallenopsisobstaculumsuperavit*	1
				*Pallenopsispatagonica*	39
				*Pallenopsispilosa*	3
				*Pallenopsisrotunda*	6
				*Pallenopsis* sp. indet.	2
				*Pallenopsisspicata*	4
				*Pallenopsisvanhoeffeni*	33
**Phoxichilidiidae**	9				
		**Anoplodactylus**	9		
				*Anoplodactylusaustralis*	9
**Pycnogonidae**	14				
		**Pentapycnon**	3		
				*Pentapycnonbouvieri*	2
				*Pentapycnoncharcoti*	1
		**Pycnogonum**	11		
				*Pycnogonumdiceros*	2
				*Pycnogonumgaini*	6
				*Pycnogonumgordonae*	3

## References

[B7575363] Arango Claudia P., Soler-Membrives Anna, Miller Karen J. (2011). Genetic differentiation in the circum—Antarctic sea spider *Nymphonaustrale* (Pycnogonida; Nymphonidae). Deep Sea Research Part II: Topical Studies in Oceanography.

[B7592397] Ballesteros Jesús A, Setton Emily V W, Santibáñez-López Carlos E, Arango Claudia P, Brenneis Georg, Brix Saskia, Corbett Kevin F, Cano-Sánchez Esperanza, Dandouch Merai, Dilly Geoffrey F, Eleaume Marc P, Gainett Guilherme, Gallut Cyril, McAtee Sean, McIntyre Lauren, Moran Amy L, Moran Randy, López-González Pablo J, Scholtz Gerhard, Williamson Clay, Woods H Arthur, Zehms Jakob T, Wheeler Ward C, Sharma Prashant P (2021). Phylogenomic resolution of sea spider diversification through integration of multiple data classes. Molecular Biology and Evolution.

[B7570817] Brenke N. (2005). An epibenthic sledge for operations on marine soft bottom and bedrock. Marine Technology Society Journal.

[B7825717] Survey British Antarctic SOMBASE PYCNOGONIDS.

[B7575211] Cano Sánchez Esperanza, López-González Pablo J. (2013). New data concerning postembryonic development in Antarctic *Ammothea* species (Pycnogonida: Ammotheidae). Polar Biology.

[B7575238] Cano-Sánchez Esperanza, López-González Pablo J. (2018). *Ammothea* species (Pycnogonida: Ammotheidae) collected during the Polarstern cruise ANT-XXIX/3 to Antarctic waters, with a description of a new species. Marine Biology Research.

[B7575261] Cano-Sánchez Esperanza, López-González Pablo J (2019). Two new species and new findings in the genus *Pallenopsis* (Pycnogonida: Pallenopsidae) with an updated identification key to Antarctic and Sub-Antarctic species.. Zootaxa.

[B7570897] Child C. A. (1994). Antarctic and Subantarctic Pycnogonida. I. The Family Ammotheidae. Antarctic Research Series.

[B7570907] Child C. A. (1994). Antarctic and Subantarctic Pycnogonida. II. The Family Austrodecidae. Antarctic Research Series.

[B7570867] Child C. A. (1995). Antarctic and Subantarctic Pycnogonida. III. The Family Nymphonidae. Antarctic Research Series.

[B7570877] Child C. A. (1995). Antarctic and Subantarctic Pycnogonida. IV. The Families Colossendeidae and Rhynchotoraxidae. Antarctic Research Series.

[B7570887] Child C. A. (1995). Antarctic and Subantarctic Pycnogonida. V. The Families Pycnogonidae, Phoxichilidiidae, Endeidae and Callipallenidae. Antarctic Research Series.

[B7575373] Collins E. E., Galaska M. P., Halanych K. M., Mahon A. R. (2018). Population genomics of *Nymphonaustrale* Hodgson, 1902 (Pycnogonida, Nymphonidae) in the Western Antarctic. The Biological Bulletin.

[B7575383] Dietz Lars, Krapp Franz, Hendrickx Michel E., Arango Claudia P., Krabbe Kathrin, Spaak Johanna M., Leese Florian (2013). Evidence from morphological and genetic data confirms that *Colossendeistenera* Hilton, 1943 (Arthropoda: Pycnogonida), does not belong to the *Colossendeismegalonyx* Hoek, 1881 complex. Organisms Diversity & Evolution.

[B7575270] Dömel Jana S., Macher Till-Hendrik, Dietz Lars, Duncan Sabrina, Mayer Christoph, Rozenberg Andrey, Wolcott Katherine, Leese Florian, Melzer Roland R. (2019). Combining morphological and genomic evidence to resolve species diversity and study speciation processes of the *Pallenopsispatagonica* (Pycnogonida) species complex. Frontiers in Zoology.

[B7570934] Fry William G., Hedgpeth Joel Walker (1969). Pycnogonida, 1: Colossendeidae, Pycnogonidae, Endeidae, Ammotheidae. New Zealand Oceanographic Institute Memoir.

[B7825664] GBIF Global Biodiversity Information Facility. www.gbif.org.

[B7570847] Gordon I. (1932). Pycnogonida. Discovery Reports.

[B7570857] Gordon I. (1944). Pycnogonida. B.A.N.Z Antarctic Research Expedition Reports.

[B7575330] Griffiths Huw J., Barnes David K. A., Linse Katrin (2009). Towards a generalized biogeography of the Southern Ocean benthos. Journal of Biogeography.

[B7575078] Griffiths Huw J., Arango Claudia P., Munilla Tomás, McInnes Sandra J. (2011). Biodiversity and biogeography of Southern Ocean pycnogonids. Ecography.

[B7575178] Hedgpeth J. W. (1969). Pycnogonida. Antarctic Map Folio Series.

[B7570837] Hodgson T. V. (1907). Pycnogonida. Natural Histoty Reports National Antarctic Expedition.

[B7570917] Horton T., Marsh L., Bett B. J., Gates A. R., Jones D. O., Benoist N., Pfeifer S., Simon-Lled&oacute E., Durden J. M., Vandepitte L., Appeltans W. (2021). Recommendations for the standardisation of open taxonomic nomenclature for image-based identifications. Frontiers in Marine Science.

[B7575395] Krabbe Kathrin, Leese Florian, Mayer Christoph, Tollrian Ralph, Held Christoph (2010). Cryptic mitochondrial lineages in the widespread pycnogonid *Colossendeismegalonyx* Hoek, 1881 from Antarctic and Subantarctic waters. Polar Biology.

[B7575188] León Tomás Munilla (2001). Synopsis of the pycnogonids from Antarctic and Subantarctic waters. Polar Biology.

[B7594774] Maxwell Jamie, Gan Yi-Ming, Van de Putte Anton, Griffiths Huw (2021). Sea spiders (Arthropoda, Pycnogonida) from ten recent research expeditions to the Antarctic Peninsula, Scotia Arc and Weddell Sea.

[B7575293] Munilla Tomás, Soler-Membrives Anna (2007). The occurrence of pycnogonids associated with the volcanic structures of Bransfield Strait central basin (Antarctica). Scientia Marina.

[B7575159] Munilla Tomás, Soler Membrives Anna (2009). Check-list of the pycnogonids from Antarctic and sub-Antarctic waters: zoogeographic implications. Antarctic Science.

[B7575302] Munilla Tomás, Soler-Membrives Anna (2015). Pycnogonida from the Bellingshausen and Amundsen seas: taxonomy and biodiversity. Polar Biology.

[B7575311] Nielsen Johanna Fønss, Lavery Shane, Lörz Anne-Nina (2009). Synopsis of a new collection of sea spiders (Arthropoda: Pycnogonida) from the Ross Sea, Antarctica. Polar Biology.

[B7826527] OBIS Ocean Biodiversity Information System. Intergovernmental Oceanographic Commission of UNESCO. www.obis.org.

[B7575340] San Vicente C., Ramos A., Jimeno A., Sorbe J. C. (1997). Suprabenthic assemblages from South Shetland Islands and Bransfield Strait (Antarctica): preliminary observations on faunistical composition, bathymetric and near-bottom distribution. Polar Biology.

[B7575320] Soler i Membrives Anna, Turpaeva Elena, Munilla Tomás (2009). Pycnogonids of the Eastern Weddell Sea (Antarctica), with remarks on their bathymetric distribution. Polar Biology.

[B7575090] Soler-Membrives A., Munilla T., Arango C., Griffiths H., Broyer C. De, Koubbi P., Griffiths H., Raymond B., d’Acoz C. Udekem (2014). Biogeographic Atlas of the Southern Ocean.

[B7575201] Stock J. H. (1957). The pycnogonid family Austrodecidae. Beaufortia.

[B7575406] Weis Andrea, Meyer Roland, Dietz Lars, Dömel Jana S., Leese Florian, Melzer Roland R. (2014). *Pallenopsispatagonica* (Hoek, 1881) - a species complex revealed by morphology and DNA barcoding, with description of a new species of *Pallenopsis* Wilson, 1881. Zoological Journal of the Linnean Society.

